# Targeted Metabolomic and Transcriptomic Analyses of “Red Russian” Kale (*Brassicae napus* var. *pabularia*) Following Methyl Jasmonate Treatment and Larval Infestation by the Cabbage Looper (*Trichoplusia ni* Hübner)

**DOI:** 10.3390/ijms19041058

**Published:** 2018-04-02

**Authors:** Yu-Chun Chiu, John A. Juvik, Kang-Mo Ku

**Affiliations:** 1 Division of Plant and Soil Sciences, West Virginia University, Morgantown, WV 26506, USA; yuchiu@mix.wvu.edu; 2 Department of Crop Sciences, University of Illinois at Urbana-Champaign, Urbana, IL 61801, USA; juvik@illinois.edu

**Keywords:** glucosinolate, methyl jasmonate, insect herbivory, kale, *Brassica* crops

## Abstract

Methyl jasmonate (MeJA), synthesized in the jasmonic acid (JA) pathway, has been found to upregulate glucosinolate (GS) biosynthesis in plant species of the *Brassicaceae* family. Exogenous application of MeJA has shown to increase tissue GS concentrations and the formation of myrosinase-mediated GS hydrolysis products (GSHPs). In vitro and in vivo assays have demonstrated the potential health-promoting effects of certain GSHPs. MeJA is also known to elicit and induce genes associated with defense mechanisms to insect herbivory in *Brassica* species. To investigate the relationship between MeJA-induced GS biosynthesis and insect defense, three treatments were applied to “Red Russian” kale (*Brassicae napus* var. *pabularia*) seedlings: (1) a 250 µM MeJA leaf spray treatment; (2) leaf infestation with larvae of the cabbage looper (*Trichoplusia ni* (Hübner)); (3) control treatment (neither larval infestation nor MeJA application). Samples of leaf tissue from the three treatments were then assayed for changes in GS and GSHP concentrations, GS gene biosynthesis expression, and myrosinase activity. Major differences were observed between the three treatments in the levels of GS accumulation and GS gene expression. The insect-damaged samples showed significantly lower aliphatic GS accumulation, while both MeJA and *T. ni* infestation treatments induced greater accumulation of indolyl GS. The gene expression levels of *CYP81F4*, *MYB34*, and *MYB122* were significantly upregulated in samples treated with MeJA and insects compared to the control group, which explained the increased indolyl GS concentration. The results suggest that the metabolic changes promoted by MeJA application and the insect herbivory response share common mechanisms of induction. This work provides potentially useful information for kale pest control and nutritional quality.

## 1. Introduction

Kale is a widely consumed leafy vegetable grown around the world. Some kale cultivars are taxonomically classified as *Brassica oleracea*, while others are classified as *Brassica napus*. The popularity of kale has recently increased, as more information has been published regarding its high nutritional value [1]. As a member of *Brassica* genus, kale leaf tissue is rich in glucosinolates (GS) which are precursors of potential health-promoting compounds [2,3,4,5]. Research results suggest that it is possible to improve GS biochemical profiles in kale production systems by changing cultivation practices and by genetic manipulation [4,6,7]. One method to potentially enhance GS accumulation in kale is through the exogenous application of the phytohormone methyl jasmonate (MeJA), similar to results observed in *Arabidopsis* and other *Brassica* crops including broccoli, cauliflower, Chinese cabbage, and pak choi [4,8,9,10,11,12]. Therefore, spray application of MeJA may be a practical and economical way to increase the nutritional quality of kale.

GS are sulfur- and nitrogen-containing plant secondary metabolites that are found in *Brassicaceae* species [13,14]. GSs can be categorized into three types, namely, aliphatic, indolyl, and aromatic GS on the basis of their respective biosynthetic precursor amino acid, i.e., methionine, tryptophan, and phenylalanine [13]. The bioactivities of GS are derived from its hydrolysis products formed by the action of the endogenous enzyme myrosinase. The hydrolysis of GS generates a wide array of GSHPs based on the the GS chemical structure, myrosinase-associated protein cofactors, pH, and the presence of Mg^2+^ and ascorbic acid [3,13,15,16,17,18,19]. GSHPs structure and concentrations have been associated with insect oviposition [20] and herbivore defense. In *Arabidopsis*, the hydrolysis products from two indolyl GS (4-methoxy glucobrassicin and neoglucobrassicin) have found to promote enhanced feeding-deterrent activity in aphids [21].

GS *in Brassicae* species provides a mechanism of defense against insect herbivory and is partially regulated by the phytohormone jasmonic acid (JA). Aliphatic and indolyl GS concentrations were found to be positively correlated with insect defense [21,22,23]. Indolyl GS biosynthesis and tissue concentrations were observed to be more sensitive to environmental variability and exogenous MeJA treatment [24,25]. JA-upregulated genes induce a majority of herbivore resistance traits in *Arabidopsis* [26,27,28], including the biosynthesis of indolyl GSs [21]. It has been suggested that JA esterase converts MeJA into JA when exogenous MeJA is applied [29]. Greater concentrations of indolyl GS were observed to enhance insect deterrence. In ‘Green Magic’ and ‘VI-158’ broccoli cultivars, the survival rate and the larval weight of cabbage looper (*Trichoplusia ni*) has been found to be significantly reduced by a 400 μM spray application of JA, attributable to the increase of neoglucobrassicin (indolyl GS) concentration [30].

Cabbage looper is a lepidopteran insect and a major pest on cole crops across many regions in the United States [31]. Cabbage looper is a generalist insect from the lepidopteran family Noctuidae, with different plant families, including *Brassiceae*, *Solanaceae*, and *Cucurbitaceae*, serving as hosts*.* As its name implies, cabbage looper is commonly observed on cabbage and other *Brassica* vegetables such as broccoli. It is a major agricultural pest not only in the United States but also in Africa and in Asia [32,33]. Because of their broad host range, cabbage loopers have evolved mechanisms to cope with different plant defense systems [34]. Therefore, this insect is an appropriate candidate to study insect herbivory in *Brassica* vegetables.

Insect herbivore-infested plants are challenged not only by physical damage: in fact, inter- and intra-cellular responses to wounding, insect oral secretions, oviposition fluids or peptides from insect’s saliva [29] promote the initiation of complex gene cascades associated with defense mechanisms. Cao’s group has found that exogenous MeJA can partially compensate in the endogenous JA-deficient *Arabidopsis* mutant *opr3* by upregulating the biosynthesis of some GS but not of the aliphatic forms [35]. In addition, jasmonate signaling is found to shape plant growth in *Arabidopsis* by modulating a transcriptional network [36] and the crosstalk with auxin signaling pathway [37]. Normally, plants allocate energy directly toward growth or reproduction, unless their defense systems are induced. Herbivore-induced defense systems also include enhancing morphological forms of defense (trichrome production [38] or thickening cell walls [39,40]) to interfere with insect feeding. To synthesize defense compounds, plants need to utilize energy or precursor compounds including sugars and amino acids [39]. Reduced sugar concentrations in response to MeJA treatment have been observed in *Brassica oleracea* and *Brassica rapa* [41,42]. It also has been suggested that a hidden transcriptional network operates to reduce growth following JA signaling in lieu of depleting energy from carbon sources [36], thus influencing vegetable eating quality. In either situation, the activation of defense mechanisms may interfere with plant primary growth [43].

Collectively, the application of MeJA sprays in agricultural production systems may result in similar physiological changes as observed under insect herbivore damage by increasing the concentration of different GS in different *Brassica* vegetables. However, the metabolic changes may not be identical to those observed under insect damage, because of temporal and spatial differences in the two scenarios. MeJA application is expected to be more homogenous than insect herbivory and to trigger a faster or more drastic metabolite change in plants. In contrast, insect herbivory may be a gradual and more complicated process, since the plants are simultaneously challenged by physical wounding and foreign peptides from the insect’s saliva. Although previous research has focused on how GS affects insect herbivory in *Arabidopsis* mutant lines or other *Brassica* crops [21,23,30,44], how exogenously applied MeJA alters metabolite concentrations and how its effects differ from those of the actual insect herbivore on plants remain unclear. To determine the potential differences in the biochemical responses, in this study, we compare the changes in GS, GSHP, and primary metabolite concentrations in plants of “Red Russian” kale undergoing insect damage (leaf tissue 4 days after infestation of *Trichoplusia ni* larvae) or MeJA spray treatment (leaf tissue harvested 4 days after 250 µM MeJA treatment).

## 2. Results and Discussion

### 2.1. Quantification of Insect-Damaged Area

The damaged leaf area after 4 days in the presence of cabbage looper larvae (second instar) was calculated via the open-source software ImageJ tool (https://imagej.nih.gov/ij/). After 4 days of insect feeding, the damaged leaf area of “Red Russian” kale from three individual plants was 12.2%, 5.1%, and 15.0%, respectively (average of 10.8%; Figure S1). We observed significant variation in GS concentrations between the apical and basal leaves of kale [4] indicating that indolyl GS concentration may be associated with cabbage looper’s growth [30]. We quantified GS from different kale leaf locations from top to bottom (Table S1 and Figure S2). According to our Table S1, younger leaves had significantly higher GS than older leaves. Therefore, the variation in GS concentrations in the different feeding locations of larvae on kale plants might impact cabbage looper’s activity, therefore producing differences in the extent of the damaged areas.

### 2.2. Effect of MeJA Application and T. ni Treatment on GS, Their Hydrolysis Products, and Myrosinase Activity

Four days after insect infestation and the application of 250 µM MeJA treatment (Sigma, St. Louis, MO, USA), treated and untreated kale plants were harvested, their leaf tissue was lyophilized, and the samples were stored at −20 °C for later analysis. A total of six GSs, including three aliphatic GSs (progoitrin, glucoraphanin, and gluconapin), and four indolyl GSs (glucobrassicin, neoglucobrassicin, 4-methoxyglucobrassicin, and 1-hydroxyglucobrassicin) were quantified by ultra-high performance liquid chromatography (UHPLC) and identified by LC mass spectrometer/mass spectrometer (Table 1; Table S2). Only trace amounts of glucoerucin and sinigrin were detected (data not shown). Kale seedlings subjected to cabbage looper feeding showed significantly lower accumulations (25.2%, *p* < 0.05) of total aliphatic GS (4.52 μmol/DW) than the control group (6.04 μmol/DW), while one-time application of 250 μM MeJA showed no difference in total aliphatic GS (6.50 μmol/DW) compared to the control group (Table 1). MeJA application on kale in the present study was consistent with an earlier report according to which the application of MeJA had no effect on aliphatic GS concentrations in *B. napus* “Red Winter” [4]. In the insect-damaged kale plants, the significantly lower concentration of aliphatic GS may result from the release of volatile hydrolysis products derived from aliphatic GS as a defense mechanism. Beekwilder and colleagues [23] found that the larval weight of the generalist lepidopteran insect *Mamestra brassicae* was 2.6-fold greater in an *Arabidopsis* mutant completely lacking aliphatic GSs. A similar result was observed [38] for cabbage looper larvae feeding on *Arabidopsis*. While the positive effects of aliphatic GS in the defense against generalist insects have been reported [45], the biosynthesis of aliphatic GS might not keep up with the loss of aliphatic GS volatile hydrolysis products. Previous work with *Brassica oleracea* has shown that aliphatic GS biosynthesis is mainly regulated by genetics (61% of total phenotypic variation) rather than by biotic and abiotic environmental factors (4.5%) [24]. Perhaps aliphatic GS biosynthesis in *B. napus* is regulated in a comparable manner, with limited response to biotic stress. This is in contrast to a previous study with *Arabidopsis*, where significantly higher accumulations of aliphatic GS were observed after a 2-day infestation with *T. ni* [46].

In the present study, the concentration of total indolyl GS was the greatest in insect-damaged kales (3.59 μmol/DW), followed by MeJA-treated kales (2.80 μmol/DW), and the control plants (0.81 μmol/DW). Indolyl GS biosynthesis has been found to be upregulated following MeJA spray applications [4,30]. However, to our knowledge, the effect of *T. ni* larval feeding on indolyl GS biosynthesis in plants has been only limited studied. Three different responses to herbivory are recognized in plant defense systems: (1) herbivore-induce immunity (HTI) associated with oviposition, (2) herbivore-associated molecular patterns (HAMPs) and damage-associated molecular patterns (DAMPs), and (3) wound-induced resistance (WIR) by mechanical wounding [47]. A previous study [46] showed the *T. ni* feeding induced high-level expression of jasmonic acid methyltransferase (*at1g19640*) and a significantly higher accumulation of indolyl GS in *Arabidopsis*. This implies that the plant response to *T. ni* infestation and the activation of the JA signaling pathway are relatively similar to those observed upon exogenous MeJA application, since methyltransferase converts JA into MeJA, and exogenous MeJA application can restore defense responses in JA signaling-impaired plants [48], and activate the JA signaling pathway as well. However, to plants, MeJA treatment may be less complex than insect damage treatment because mechanical wounding by insects and foreign peptides from the insect’s saliva is lacking when applying exogenous MeJA. Considering this aspect, gene expression patterns altered by insect damage and by MeJA treatment may vary and thus lead to the difference in GS accumulation observed after the two treatments.

Myrosinase-mediated hydrolysis products, especially isothiocyanates from GS, have been found to interfere with the growth and development of generalist herbivores, while providing oviposition cues for specialists [49,50,51]. In this study, we detected seven hydrolysis products from aliphatic GS (sulforaphane, sulforaphane nitrile, 3-butenyl isothiocyanate, 1-cyano-3,4,-epithiobutane, crambene, goitrin, and 1-cyano-2-hydroxy-3,4-epithiobutane) and three hydrolysis products from indolyl GS [*N*-methoxyindolyl-3-carbinol (NMI3C), *N*-methoxyindolyl-3-carboxyaldehyde (NM3CA), and indolyl-3-acetonitrile (I3A)] from kale leaf samples (Table 2). In most cases, the concentrations of the hydrolysis products showed no differences between the control and the treated samples, except for the concentration of 1-cyano-3,4,-epithiobutane, which was significantly lower in insect-damaged samples than in the control and MeJA-treated kale. Sulforaphane, goitrin, NMI3C, 1M3CA, and I3A were not detected in control plants. In insect-treated kales, GSHPs were mainly converted to isothiocyanate rather than to nitrile or epithionitrile forms, and this can be related to the function of GSHPs in plant herbivory defense function, which also may lead to differences between MeJA treatment and insect treatment. A previous study [52] showed that isothiocyanates play a main role in plant defensive mechanism because of their universal toxicity; therefore, the higher accumulation of isothiocyanates in insect-damaged kales observed in the present study could be a strategy to repel *T. ni* infestation.

The concentrations of the hydrolysis products across biological replicates of insect-damaged samples varied greatly, while the variation of the hydrolysis products among MeJA-treated plants remained small. The coefficient of variance (CV) across all 10 hydrolysis products from insect-damaged samples ranged from 34% to 136%, while the CV of MeJA-treated samples was relatively small (10% to 27%). These results suggest that the stimulus from the insect herbivory process might be more dynamic than the one-time MeJA application, because insects can be exposed to significantly different levels of GS depending on kale leaf location and respond accordingly (Table S1).

### 2.3. Effect of MeJA Application and T. ni Larva Feeding on the Expression of Genes Related to Indolyl GS Biosynthesis and of Indolyl GS Transcription Factors, Myrosinase, and Specifier Proteins

**Figure 1:**
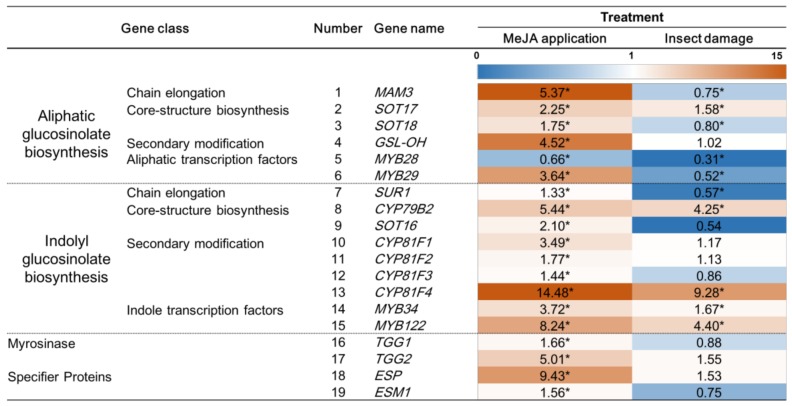
Expression of known genes involved in the GS biosynthesis pathway, myrosinase biosynthesis, and related to specifier proteins associated with GS hydrolysis in extracts of “Red Russian” kale plants 4 days after treatment with 250 μM MeJA or insect damage. Asterisk indicates a significant difference compared to the control using student *t*-test (*p* < 0.05). The values highlighted in red and in blue indicate significantly upregulated or downregulated, respectively.

To understand the different effects of exogenous 250 μM MeJA application and *T. ni* larval feeding on the expression of genes related to GS biosynthesis or their hydrolysis products in “Red Russian” kale, we investigated gene expression by quantitative real-time polymerase chain reaction (qRT-PCR) to measure the abundance of transcripts associated with the above-mentioned pathways under the two treatments. We measured six genes involved in the aliphatic GS biosynthesis pathway, including *MAM3*, *SOT17*, *SOT18*, *GSL-OH*, *MYB28*, and *MYB29*, and nine genes involved in indolyl GS biosynthesis, including *SUR1*, *CYP79B2*, *SOT16*, *CYP81F1*, *CYP81F2*, *CYP81F3*, *CYP81F4*, *MYB34*, and *MYB32* (Figure 1; Table S3). In addition to the GS biosynthetic genes, genes involved in GS hydrolysis, including myrosinase-encoding genes *TGG1* and *TGG2*, epithiospecifier protein (*ESP*), and epithiospecifier modifier 1 (*ESM1*), were measured to investigate the potential changes in the levels of hydrolysis products (Figure 1; Table S2).

Among these genes, exogenous 250 μM MeJA application significantly upregulated the aliphatic GS-related genes *MAM3* (chain elongation), *SOT17* (core biosynthesis), *SOT18* (core biosynthesis), *GSL-OH* (secondary modification), *MYB29* (transcription factor), *TGG1* and *TGG2*, *ESP*, and *ESM1*, between 1.7- and 9.4-fold. *T. ni*-induced damage only significantly upregulated *SOT17,* while there was a significant downregulation of *MAM3*, *SOT17*, *SOT18*, *MYB28*, and *MYB29*. These results indicated that a spray application of 250 μM MeJA is sufficient to induce the majority of aliphatic GS biosynthesis genes in *B. napus* kale. It has been reported that a 400 μM MeJA application to the heads of the broccoli cultivar “Green Magic” can significantly increase *SOT17*, *SOT18*, *TGG1*, *TGG2*, and *ESM1,* but this is not observed in the doubled haploid inbred “VI-158” [30]. Noticeably, the above genes were not significantly upregulated by a 200 μM MeJA spray application in the previous study using ‘Green Magic’ and “VI-158”. The effect of exogenous MeJA application on aliphatic GS gene expression varies depending on the application concentration and on plant genotype. *MYB28* was the only gene that was significantly downregulated by the 250 μM MeJA treatment. Different gene homologues encoding *MYB28* may have differential sensitivity to exogenous MeJA, since it was reported that these genes can be differentially expressed in allopolyploid *Brassica juncea* [53]. Yi’s study [54], that applied 250 μM MeJA to *B. oleracea* kale, found that the relative gene expression of *MYB28* was inconsistent among all measured homologues encoding *MYB28.* The authors reported that a 250 μM MeJA application significantly increased, decreased, or did not change the levels of *MYB28* transcripts. The transcript we measured was Bol036286, and our result agrees with their results regardless of the fact that different species were used in the two studies. Another possible reason is that the primers used in *B. oleracea* did not bind or effectively amplify the transcripts of *MYB28*. *B. napus* kale is an allopolyploid which contains the genome from *B. rapa* (AA) and *B. oleracea* (CC) and many gene duplications, and subsequent functional divergence has occurred during polyploidization [55]. Thus, *MYB28* homologs might not have been accurately measured when we analyzed the data. Interestingly, even with increased gene expression levels, the total aliphatic GS concentration in MeJA-treated kale was not significantly greater compared to control kale samples (Table 1). This suggests that the gene expression patterns were not tightly associated with changes in aliphatic GS concentrations. In contrast, *T*. *ni* larval feeding on “Red Russian” kale did not upregulate aliphatic GS genes like MeJA treatment and significantly reduced the relative gene expression of *MAM3*, *SOT17*, *SOT18*, *MYB28*, and *MYB29* (Figure 1). The gene expression pattern in the *T. ni-*treated samples was associated with variations in aliphatic GS concentrations (Table 1), with reduced gene expression resulting in significant lower aliphatic GS concentrations. This suggests that the effect of four-day feeding of *T. ni* on aliphatic GS gene expression in “Red Russian” kale could be transient or that plant samples need to be harvested after fewer days of Please check if the original meaning is retained. Mewis et al. [56] conducted an insect feeding study on the lepidopteran generalist *Spodoptera exigua* Hübner on *Arabidopsis* and reported *MAM3* was significantly upregulated after only one day of larval feeding.

Indolyl GS and its hydrolysis products have been found to be strongly associated with insect herbivory [29,49]. In the present study, a 250 μM MeJA application led to a significantly increased (*p* < 0.05) expression in “Red Russian” kale of multiple genes related to indolyl GS biosynthesis, such as *CYP79B2* (core biosynthesis), *SOT16I* (core biosynthesis), and *CYP81F1*, *CYP81F2*, *CYP81F3*, and *CYP81F4* (side chain modification), and the transcription factors *MYB34* and *MYB122* (Figure 1). Only the transcript levels of *CYP79B2*, *CYP81F4*, *MYB34*, and *MYB122* were found to significantly increase in *T. ni* larval-treated kale samples (Figure 1).

*MYB34* has been recognized as an activator in the indolyl GS biosynthesis pathway [57] and after exogenous MeJA application in *Arabidopsis* [58], pak choi (*Brassica rapa* ssp. *chinensis*) [59], and two different broccoli cultivars (*Brassica oleracea* ssp. *italica*) [30]. Our findings are comparable to those of these previous studies where greater expression of *MYB34* in MeJA-treated kale resulted in a significantly higher accumulation of indolyl GS (Table 1). *CYP79B2* is an early upstream gene in the indolyl GS biosynthesis pathway that functions to convert tryptophan into indolyl-3-acetaldoxime [60]. This conversion is also the first step in the biosynthesis of the plant hormone auxin [61]. The interconnection between auxin homeostasis and the JA signaling pathway has been previously reported in *Arabidopsis* [62]. The higher expression of *CYP79B2* in MeJA-treated kale from the present study agrees with results observed in *Arabidopsis*. We also detected higher expression levels of *CYP81F1*, which is involved in indolyl GS side chain modification in MeJA-treated kale functions to convert glucobrassicin into 4-hydroxyindol-3-ylmethy GS. *CYP81F1* has been reported to be induced by exogenous MeJA application in broccoli, kale, and cabbage [54]. In Yi et al. [54], the significantly increased *CYP81F1* expression levels resulting from a 250 μM MeJA spray application to *B. oleracea* kale provided a result to similar ours. It is important to consider the difference in species response to exogenous MeJA application, which can be dose-dependent and cultivar-specific [30]. The higher expression levels of *CYP81F1* may also be associated with the significantly greater accumulation of 4-methoxy glucobrassicin (Table 1). The gene expression of *TGG2* and *ESP* in the MeJA-treated group showed a 5-fold and 9.5-fold significant increase when compared to the control kale. *ESP* is a myrosinase-associated protein involved in catalyzing the formation of epithionitriles or simple nitriles during GS hydrolysis, depending on the structure of GS [63]. *ESP* promotes the formation of epithionitriles over other hydrolysis products, such as isothiocyanates, resulting in weaker human anti-cancer bioactivity involving the induction of phase II detoxification enzymes in Hepa1c1c7 cell cultures [64]. Isothiocyanates are also the favored form of GSHPS that have insect repellent activity [65], and were associated with lower gene expression levels of *ESP* in *T. ni*-treated kale.

In the present study, insect-damaged kale did not show the same pattern of gene expression, suggesting that larval feeding was not perceived or did not provide as intense a response as observed following exogenous application of 250 μM MeJA in kale plants. It is possible that the intensity of the response caused by six-second instar of *T. ni* feeding on “Red Russian” kale plants was not strong enough to induce the expression of *CYP79B2*. However, considering the indolyl GS changes in Table 1, we suggest that the peak increase of gene expression in insect-damaged kale was too transient or that mRNA transcript turnover had reduced the transcripts' levels when the kale samples were harvested for the analyses [30,56,66]. *T. ni* damage may be more location-specific than MeJA treatment and, therefore, it might not be as powerful as MeJA application in triggering gene regulation. As a generalist insect, *T. ni* is generally exposed to a broad array of plants. The chemical composition of *T. ni*’s saliva can be altered when *T. ni* confronts different plant species, as suggested by a recent study [34]. Therefore, it is likely that the damage by *T. ni* was not as effective as the MeJA treatment in activating genes involved in the indolyl GS biosynthesis pathway. Noticeably, the expression levels of *MYB34*, *CYP79B2*, and *CYP81F1* in insect-damaged were slightly higher than those in control kale, but not significantly different. Compared to the previous exogenous MeJA application study, significantly increased levels of gene expression were measured 2 days after MeJA application on broccoli [67] and pak choi [59], or three days after treatment on broccoli [30].

### 2.4. Effect of MeJA Application and T. ni Larval Feeding on Myrosinase Activity and Nitrile Formation

**Figure 2:**
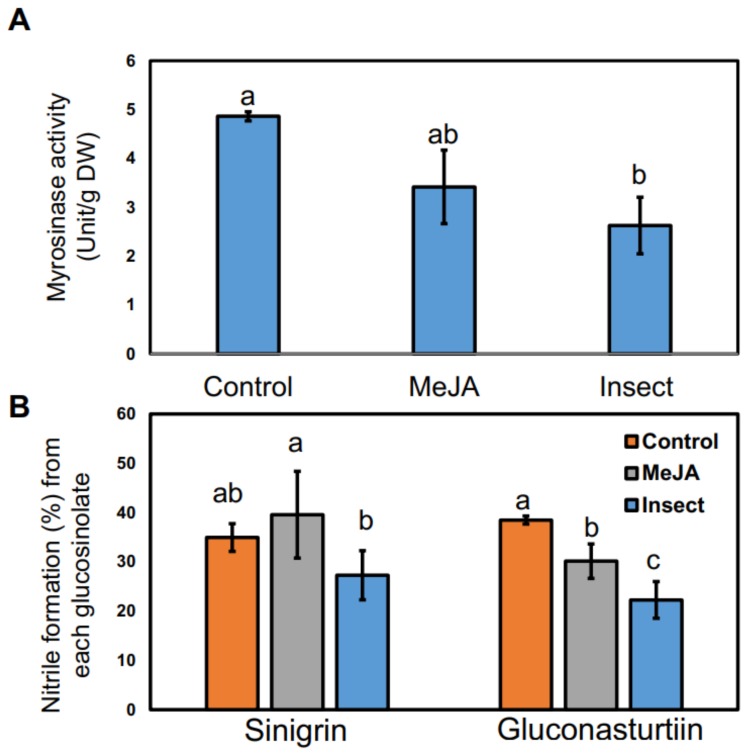
(**A**) Myrosinase activity (Unit/g DW) and (**B**) Nitrile formation (%) of control, insect-damaged, and MeJA-treated kale. Nitrile formation (%) is shown as the relative ratio of nitrile to the total concentration of the hydrolysis products formed (sum of isothiocyanates and nitriles) from sinigrin and gluconasturtiin. The data are presented as the mean concentration ± standard error (*n* = 3). Different letters mean significantly different by Student’s *t*-test (*p* < 0.05) across three groups on (a) and three groups within precursor GS on (b), respectively.

The greatest myrosinase activity was detected in control samples (4.86 ± 0.16 Unit/g DW), while reductions in the activity were observed in both MeJA-treated samples (3.42 ± 1.00 Unit/g DW) and insect-damaged samples (2.63 ± 1.31 Unit/g DW), with 30% and 45% lower activity than in the control, respectively (Figure 2A). In general, myrosinase activity can be influenced by insect herbivory; however, different responses related to myrosinase post-translational glycosylation, myrosinase complexation with associated proteins, or the levels of the cofactor ascorbate were observed after treatment with specialist or generalist insects [68]. Decreased myrosinase activity from insect-damaged kale in this study may result from the harvesting regime (four days after feeding initiation). Martin’s group conducted a feeding experiment with the generalist insect *Athalia rosae* on *Sinapis alba* (Family *Brassicaceae*) and found a strong increase in myrosinase activity in day-one tissue samples, decreased activity in day-two samples, and no difference in control and insect-treated samples on day four after feeding initiation [69]. The effects of MeJA application on myrosinase activity differed among the species tested (broccoli or kales) and between leaf tissue samples at different developmental stages (apical or basal leaves). Additionally, a reduction of transcript abundance of myrosinase and its cofactors was observed in broccoli four days after 500 μmol MeJA application [4,67]. This suggests that there can be rapid changes in myrosinase activity in response to herbivory or MeJA application.

Nitrile formation (%) from sinigrin was found to be increased only by MeJA treatment. Both MeJA spray application and larval feeding significantly decreased nitrile formation following hydrolysis of gluconasturtiin (Figure 2B). The concentration of nitriles formed indirectly reflects the activity of ESP which promotes epithionitriles as hydrolysis products from alkenyl GS [19], or nitriles from other GS [70]. Isothiocyanates can be used as cues for host recognition by specialist herbivores. Generalist herbivores like *T. ni* were found to show feeding preference for nitrile-producing *Arabidopsis* lines [70]. Hydrolysis products from insect-damaged kale comprised lower concentrations of isothiocyanates.

### 2.5. Effect of MeJA Application and T. ni Treatment on Polar Primary Metabolites

In addition to the change of secondary metabolites under the various treatments, changes in several primary metabolite concentrations (sugars, organic acid, sugar alcohol, and amino acids) were measured via gas chromatography–mass spectrometry (GC–MS) to further illustrate the effects of insect damage and MeJA treatment on kale plants.

**Figure 3:**
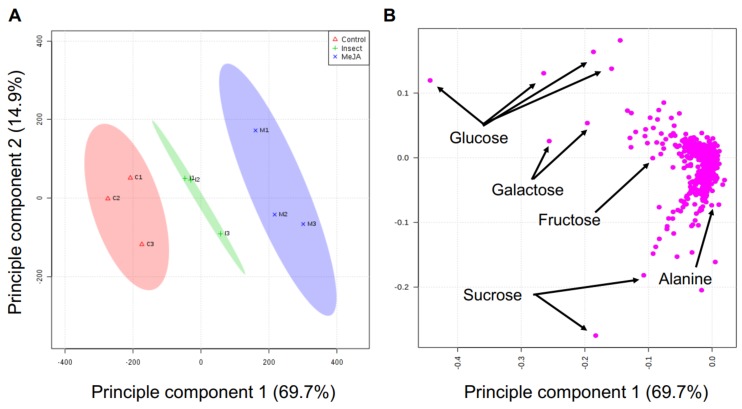
Principle component analysis (**A**) score and (**B**) loading plots derived from non-targeted metabolite profiling of *Brassica napus* with the control plants, MeJA-treated plants, and insect-feeding plants. The shaded areas in (**A**) represents 95% confidence regions. The pink dots indicated by arrows in (**B**) represent discriminating biomarkers among various treatments.

Principal component analysis (PCA) was utilized to compare the changes in metabolites and identify significantly differences in metabolites between control and treatment groups. According to PCA scores plot, the distribution of metabolites in MeJA-treated and insect-damaged kale was clearly different compared to the control (Figure 3). Principal component 1 (PC1) mainly analyzed the treatment effects and accounted for 69.7% variation among the three treatments, whereas PC2 (14.9%) accounted for variations of the biological replicates within each treatment. The loading plot shows that the variables are correlated with PC1 and PC2 (Figure 3).

**Figure 4:**
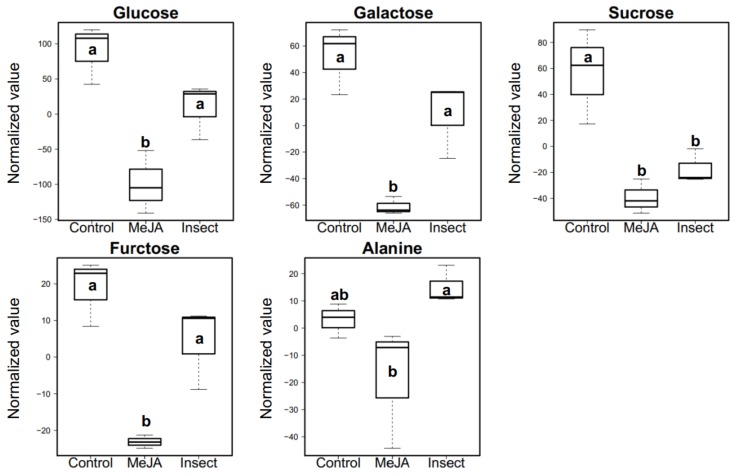
Five biomarker metabolites selected with top-5 variable importance in projection (VIP) scores (VIP > 1.2) from all metabolites among control kales, MeJA-treated kales, and insect-infected kales. Different letter indicates significant differences between the groups, determined by Student’s significance test (*p* < 0.05). The presented values are normalized values based on an internal standard with auto-scale in Metaboanalyst.

The most significantly different concentrations in metabolites among the three treatments were selected by partial least-squares discriminant analysis (PLS-DA) using variable importance in projection (VIP) to estimate the importance of variables in the model. VIP score 1.2 was set as a threshold. Glucose, galactose, sucrose, fructose, and alanine (Figure 4) were above VIP score 1.2, which indicated that these compounds are important biomarkers that explain the concentration differences among treatments (Figure 4). Overall, sugar concentrations were decreased in both the MeJA and insect feeding treatments, suggesting that plants utilize mono- and disaccharide sugars as a carbon source to synthesize GSs or other chemicals for defense. Carbohydrates and amino acids are the primary metabolites that are reported to change under conditions of insect herbivory in plants. It has been reported that leaf sugar levels are regulated by JA signaling in *Nicotiana attenuata* plants at various developmental stages [71]. Mechado and colleagues [71] presented evidence that the concentrations of glucose, fructose, and sucrose in plants were inversely correlated with endogenous JA concentrations. Alanine was the only amino acid whose concentration differed among the treatment groups (VIP score = 1.4). MeJA treatments have been reported to change the levels of sugars, organic acids, amino acids, and certain GSs in *Brassica* crops [41,42]. The changes in these primary metabolites were found cutivar-specific in pak choi [42]. Mono- and disaccharide sugar concentrations were decreased by MeJA spray treatment in “Red Russian” kale, as previously reported [42]. Sugars such as sucrose play a pivotal role in generating metabolic energy and provide a range of physiological functions in plant respiration [72]. The mono- and disaccharide sugar concentrations in insect-damaged kale were significantly higher than in MeJA-treated kale (Figure 4). This is the most notable difference in the metabolomes of MeJA-treated and insect-treated kale. Decreased photosynthetic activity in plants undergoing herbivory is common, since sources of carbon are needed to produce the defensive compounds. The jasmonic acid signaling pathway served to trigger these responses [73,74]. In this experiment, larvae-infested kale had a proportion of healthy leaves actively involved in photosynthesis.

## 3. Materials and Methods

### 3.1. Kale Cultivation and Treatments

The kale variety used in this experiment was “Red Russian” (Johnny’s Selected Seeds, Winslow, ME). Seeds of “Red Russian” kale were germinated in 32-cell plant plug trays filled with Sunshine^®^ LC1 (Sun Gro Horticulture, Vancouver, BC, Canada) professional soil mix. The plants were grown in a greenhouse at the University of Illinois at Champaign-Urbana at 25°C/18°C and 14 h–10 h day–night temperature regime, with supplemental high-intensity discharge (HID) lighting and 50–70% relative humidity. Four weeks later, the plants in the vegetative growth stage were transferred to 1 L pots in the greenhouse under the same conditions. Kale plants with eight fully developed leaves were selected for the experiments.

The control plants were sprayed with a 0.1% Triton X-100 (Sigma-Aldrich, St. Louis, MO, USA) solution. MeJA-treated plants were sprayed with a solution of 250 µM JA (Sigma-Aldrich, St. Louis, MO, USA) with 0.1% Triton X-100. MeJA-treated kale leaf samples were harvested four days after spray treatment. Using a paintbrush, insect-treated plants were infested with second-instar larvae of *T. ni* (Hübner). *T. ni* was cultured in the Department of Entomology at the University of Illinois Urbana-Champaign. Six larvae of *T. ni* per plant were employed, with three plants per treatment, and the leaf tissue was scored for damage and then harvested for the analyses after four days of feeding.

### 3.2. Quantification of Insect-Damaged Area Using ImageJ

Image Processing and damaged leaf area calculation were carried out via an open-source software ImageJ (https://imagej.nih.gov/ij/). (Figure S1). Area selection was performed by “wand tool” or “Freehand selection” to trace the desired area. Area calculation was based on the selected area and calculated by the software by extracting pixels information from a scale of the actual ruler in the taken photo.

### 3.3. Quantification of Glucosinolate Concentrations

All above-ground leaves of control, insect-infested, and MeJA-sprayed kale plants were harvested after four days of treatment for GS analysis. Above-ground aerial leaf and stem samples were frozen in liquid nitrogen and stored at −20°C prior to freeze-drying. Freeze-dried tissues were ground into a fine powder using a coffee grinder and stored at −20°C prior to GS analysis using high-performance liquid chromatography (HPLC). Extraction and quantification of GSs using HPLC were performed using a previously published method [75]. An amount of 200 mg freeze-dried kale leaf and stem powder and 2 mL of 70% methanol were added to 10 mL tubes (Nalgene) and heated on a heating block at 95°C for 10 min. After cooling on ice, 0.5 mL benzylglucosinolate (1 mM) was added as an internal standard (POS Pilot Plant Corp, Saskatoon, SK, Canada), mixed, and centrifuged at 8000× *g* for 5 min at 4°C. The supernatant was saved, and the pellet was re-extracted with 2 mL 70% methanol at 95°C for 10 min, after which the two extracts were combined. A subsample (1 mL) from each pooled extract was transferred into a 2 mL microcentrifuge tube (Fisher Scientific, Waltham, MA, USA). The proteins were precipitated with 0.15 mL of a 1:1 mixture of 1 M lead acetate and 1 M barium acetate. After centrifuging at 12,000× *g* for 1 min, each sample was loaded onto a column containing 1M NaOH and 1M pyridine acetate-charged DEAE Sephadex A-25 resin (GE Healthcare, Piscataway, NJ, USA) for desulfation with arylsulfatase (*Helix pomatia* Type-1, Sigma-Aldrich, St. Louis, MO, USA) for 18 h, and the desulfo-GSs were eluted with 3 mL Millipore-filtered ddH2O. The samples (100 µL) were injected onto an Agilent 1100 HPLC system (Agilent, Santa Clara, CA, USA), equipped with a G1311A binary pump, a G1322A vacuum degasser, a G1316A thermostatic column compartment, a G1315B diode array detector, and an HP 1100 series G1313A autosampler. The UV detector was set at 229 nm wavelength. An all-guard cartridge pre-column (Alltech, Lexington, KY, USA) and a Kromasil RP-C18 column (250 mm × 4.6 mm, 5 µm particle size, Supelco, Bellefonte, PA, USA) were used for quantification. The flow rate was 1 mL/min with mobile phase A (water *w*/1% acetonitrile *v*/*v* and 1 mM ammonium acetate) and B (100% acetonitrile) with the following elution profile: 0 min 0% B, 7 min 4% B, 20 min 20% B, 35 min 25% B, 36 min 80% B, 40 min 80% B, 41 min 0% B, and 50 min 0% B. Benzylglucosinolate was used as an internal standard for quantification whose relative response factor (RRF) was set as 1 [76]. The UV response factors for various glucosinolates [77,78,79] applied for quantification in this experiment were: glucoiberin 1.07, progoitrin 1.09, glucoraphanin 1.07, sinigrin 1.00, gluconapin 1.11, glucoerucin 1.00, glucobrassicin 0.29, 4-hydroxyglucobrassicin 0.28, 4-methoxyglucobrassicin 0.25, gluconasturtiin 0.95, and neoglucobrassicin 0.20 [80]. RRF of 1-hydroxyglucobrassicin was set as 0.28, just like that of 4-hydroxyglucobrassicin because RRF was not available.

The identification of desulfo-GS was based on Capriotti et al. with a slight modification [81], and using fragmentation diagnostic ions from Kusznierewicz’s group [79] (Table S2), the profiles were validated by LC-tandem MS using a Waters 32 Q-Tof Ultima spectrometer coupled to a Thermo Accela 1200 UHPLC system coupled to a heated ESI source and to a Q Exactive high-resolution (HR) quadrupole and orbitrap LC-MS/MS (Thermo Scientific, Waltham, MA, USA), operated using full scan and a parallel reaction monitoring (PRM) mode. A 250 mm × 2.1 mm with internal diameter 5 μm, 100 Å, Kromasil RP-C18 column was used (AkzoNobel, Bohus, Sweden) for extract separation. Deionized distilled water with 0.1% formic acid (mobile phase A) and acetonitrile with 0.1% formic acid (mobile phase B) were used with a gradient system: 0 min 1.5% B, 2 min 8% B, 15 min 30% B, 16 min 100% B, 25 min 100% B, 25.1 min 2% B, 30 min 2% B, with a flow rate of 0.5 mL min^–1^. Ten microliter of desulfo-glucosinolate extract was injected. The column was heated to 40°C with a column heater. Mass identification was acquired in both positive and negative ion mode. Full scan mode range was *m*/*z* 100.0–900.0. Nitrogen gas was used for ESI, collision Resolution was 70,000 FWHM (at *m/z* 200). Automatic gas control (AGC) value was 3×10^6^ in full scan; maximum ion inject time was 200 ms. The ion spray voltage was 3.9 kV with capillary temperature at 320°C. Aux gas flow rate was S-Len, RF level was 55. MS/MS isolation window width was 4.0 *m*/*z*, with resolution at 17,500 in both positive and negative modes. AGC value was 2×10^5^, and maximum ion inject time was 100 ms. 

### 3.4. Quantification of Glucosinolate Hydrolysis Products

Freeze–dried kale powder (50 mg) was suspended in 1 mL distilled water in a 2 mL microcentrifuge tube (Fisher Scientific, Waltham, MA, USA). Hydrolysis products were generated naturally by endogenous myrosinase in the absence of light at room temperature for 24 h. After adding 1 mL of dichloromethane, the samples were centrifuged at 12,000× *g* for 2 min, and the lower organic layer was collected. A gas chromatograph (Trace 1310 GC, Thermo Fisher Scientific, Waltham, MA, USA) coupled to an MS detector system (ISQ QD, Thermo Fisher Scientific, Waltham, MA, USA) and an autosampler (Triplus RSH, Thermo Fisher Scientific, Waltham, MA, USA). A capillary column (DB-5MS, Agilent Technologies, Santa Clara, CA, USA; 30 m × 0.25 mm × 0.25 µm capillary column) was used to determine GS hydrolysis products. A 1 μL sample of the dichloromethane extract was injected into the GC–MS with a split ratio of 1:1. After an initial temperature held at 40°C for 2 min, the oven temperature was increased to 260°C at 10°C/min and held for 10 min [42,82]. The injector and detector temperatures were set to 200°C and 280°C, respectively. The flow rate of the helium carrier gas was set to 1.1 mL/min. The peaks were identified using information from a previous publication [42] or by comparison with data in the National Institute of Standards and Technology (NIST) library.

### 3.5. Measurement of Myrosinase Activities and Nitrile Formation

Myrosinase activity was estimated as the total amount of hydrolysis products produced within 60 min [82]. One unit was defined as 1 μmol of the above four hydrolysis products released per min. Nitrile formation in the sample was determined by incubating concentrated horseradish root extract with protein extracts of kale. The horseradish extract was used as an exogenous substrate source of sinigrin and gluconasturtiin at the saturated level in order to minimize the reaction of kale proteins with endogenous GS substrates. Subsequently, only the hydrolysis products from sinigrin and gluconasturtiin were the dominant compounds detected by GC–MS.

Freeze–dried kale powder (75 mg) was mixed with 1.5 mL of concentrated “1091” horseradish root extract [10] in 2 mL microcentrifuge tubes (10 g of horseradish was mixed with 100 mL of 70 % methanol. This solution was centrifuged at 4000 × *g* for 5 min. The supernatant of the horseradish root extracts was transferred to a beaker and boiled until the solvent was evaporated and then it was reconstituted with 50 mL of deionized water). After centrifugation at 12,000× *g* for 2 min, 0.6 mL of supernatant was transferred to a 1.5 mL Teflon centrifuge tube (Savillex Corporation, Eden Prairie, MN, USA), and then 0.6 mL of dichloromethane was added. The tubes were placed upside down to minimize the loss of volatile compounds at room temperature for 10 min. Then, the tubes were vortexed and centrifuged at 12,000× *g* for 4 min. The dichloromethane organic layer was injected into a GC–MS (Trace 1310 GC, Thermo Fisher Scientific, Waltham, MA, USA) coupled to an MS detector system (ISQ QD, Thermo Fisher Scientific, Waltham, MA, USA) and an autosampler (Triplus RSH, Thermo Fisher Scientific, Waltham, MA, USA). A capillary column (DB-5MS, Agilent Technologies, Santa Clara, CA, USA; 30 m × 0.25 mm × 0.25 µm capillary column) was used to determine GS hydrolysis products. After an initial temperature held at 40°C for 2 min, the oven temperature was increased to 320°C at 15°C/min and held for 4 min. Injector and detector temperatures were set to 270°C and 275°C, respectively. The flow rate of the helium carrier gas was set to 1.2 mL/min. Standard curves of allyl isothiocyanate, 2-phenthyl isothiocyanate, and 3-phenylpropionitrile (Sigma-Aldrich, St Louis, MO, USA) were used for quantification. A standard curve generated from allyl isothiocyanate was applied to quantify of 1-cyano-2,3-epithiopropane.

### 3.6. RNA Extraction and Quantitative Real-Time PCR

Total RNA was isolated from control and insect- MeJA-treated freeze–dried kale powder samples using the RNeasy Mini Kit (QIAGEN), according to the manufacturer’s instructions. The quantity of RNA was measured using a NanoDrop 3300 spectrophotometer (Thermo Scientific, Waltham, MA, USA). One μg of total RNA was reverse-transcribed with Superscript™ III First-Strand Synthesis SuperMix for qRT-PCR (Invitrogen, Carlsbad, CA, USA), according to the manufacturer’s instructions. The resulting cDNA samples were diluted to 1/10 of their concentrations (*v*/*v*) for qRT-PCR. The primer sets of GS biosynthesis genes, hydrolysis genes, and transcription factor genes were designed on the basis of database-published sequences (http://www.ocri-genomics.org/bolbase/index.html) [83]. A final list of the primers used, the gene model from which they were created, and a classification of the gene can be found in Table S3. The qRT-PCR data were expressed after normalization to the broccoli actin gene (*BoACT1*) [41]. The primers were synthesized by Integrated DNA Technologies (Coralville, IA, USA). Quantitative real-time PCR was carried out with the Power SYBR^®^ Green RT-PCR Master Mix (QIAGEN) using an ABI 7900HT Fast Real-Time PCR System (Applied Biosystems, Foster City, CA, USA) according to the manufacturer’s instructions. The relative expression ratio was determined with the equation 2^−∆∆*C*t^, using the *BoACT1* normalized ∆*C*_t_ values generated by the ABI 7900HT Sequence Detection System Software 2.4 (Applied Biosystems) [67].

### 3.7. Untargeted Metabolomics by GC–MS

Primary metabolites were extracted by published protocol [75] with modifications on the extraction solvent volume. The samples (50 mg) were weighed in 2 mL microcentrifuge tubes, followed by addition of 80 μL of ribitol (10 mg/mL) as an internal standard, then extracted with 1.4 mL of methanol at 75°C. After cooling, the sample extracts were centrifuged at 15,000× *g* for 3 min, and 0.7 mL of supernatants was transferred to new 2 mL microcentrifuge tubes. To fractionate polar compounds, 0.375 mL of cold chloroform (−20 °C) and 0.7 mL cold water (4°C) were added. After vigorous mixing, the extracts were centrifuged at 15,000× *g* for 3 min, and then 50 μL supernatant was transferred to 1.5 mL microcentrifuge tubes. The extracts were dried using Vacufuge^TM^ concentrator (Eppendorf^TM^, Thermo Fisher Scientific, Waltham, MA, USA) with 10 μL of methanol to facilitate water evaporation. The dried extracts were derivatized with 50 μL of methoxyamine hydrochloride (40 mg/ml in pyridine) for 90 min at 37°C, then with 70 μL MSTFA + 1%TMCS at 37°C for 30 min. The metabolites were analyzed using a GC–MS (Trace 1310 GC, Thermo Fisher Scientific, Waltham, MA, USA) coupled to an MS detector system (ISQ QD, Thermo Fisher Scientific, Waltham, MA, USA) and an autosampler (Triplus RSH, Thermo Fisher Scientific, Waltham, MA, USA). A capillary column (Rxi-5Sil MS, Restek, Bellefonte, PA, USA; 30 m × 0.25 mm × 0.25 µm capillary column w/10 m Integra-Guard Column) was used to detect polar metabolites. After an initial temperature held at 80°C for 2 min, the oven temperature was increased to 330°C at 15°C/min and held for 5 min. The injector and detector temperatures were set to 250°C and 250°C, respectively. An aliquot of 1 μL was injected with a split ratio of 70:1. The helium carrier gas was kept at a constant flow rate of 1.2 mL/min. The mass spectrometer was operated in positive electron impact mode (EI) at 70.0 eV ionization energy at *m*/*z* 40–500 scan range.

The acquired chromatograms were converted to mzXML using the RawConverter [84]. Peak detection and alignment were performed by XCMS package in R language with default settings [85]. All data were normalized to unique ion peak (319) from the internal standard in the online platform MetaboAnalyst [86], and further statistical analysis was conducted after Pareto scaling. Metabolite identification was based on standard compounds (STD) in comparison with the mass spectra present in The National Institute of Standards and Technology (NIST) and retention times (Table S4).

## 4. Conclusions

This paper demonstrated how 250 uM MeJA spray applications and *T. ni* larval feeding affected “Red Russian” kale GS profiles, hydrolysis products formation, related gene expression profiles, and primary metabolites production. In general, both treatments significantly triggered the accumulation of GS. MeJA treatment induced higher accumulation of aliphatic GSs, while the insect feeding treatment induced higher accumulation of indolyl GSs (Table 1). Increased gene transcript abundance in the GS biosynthetic pathway was primarily observed in MeJA-treated kale (Figure 1). Both treatments were shown to increase the hydrolysis products produced from the aliphatic or indolyl GS pathways compared to the control group (Table 2), but there was no distinguishable difference in terms of hydrolysis product concentrations between the MeJA application and the insect feeding treatment. Gene expression of myrosinase, the enzyme responsible for the hydrolysis of GS and the expression of transcripts of the specifier proteins *ESP* and *ESM1* were significantly higher only in the MeJA-treated kale. Although MeJA-treated kale had slightly higher nitrile formation than control kale, insect damaged-kale had significantly lower nitrile formation than control kale. Therefore, with a better understanding of the regulation mechanism of *ESP* or *ESM1* in the presence of insect damage, it could be useful to enhance isothiocyanate production from GS. We also detected differences in the concentrations of four sugars (glucose, galactose, sucrose, and fructose) and one amino acid (alanine) between control and treatment groups. This variation is likely due the production of defense-related compounds associated with insect herbivory and the induction of the jasmonic acid pathway. The ideal sample size was suggested to be 1:10 [87,88] for PCA, so undoubtedly the statistical power was not ideal because of the small sample size. Yet, this study still suggests the potential utility of MeJA application in “Red Russian” kale to prime plants for enhanced insect defense by eliciting greater accumulations of GS and of its hydrolysis products. This study also suggests that MeJA spray treatment potentially enhances the health-promoting properties of kale by increasing the concentrations of GS and GS hydrolysis products [3].

## Figures and Tables

**Table 1:** Glucosinolate profiles (μmol·g^-1^ DW) of “Red Russian” controls and kale plants treated with 250 μM methyl jasmonate (MeJA) or infested with cabbage looper for 4 days ^y^.

Samples	Glucoraphanin	Gluconapin	Progoitrin	Glucobrassicin	Neoglucobrassicin	4-Methoxy-glucobrassicin	1-Hydroxy-glucobrassicin	Total Aliphatic GS ^z^	Total Indolyl GS	Total GS
Control	1.14 ± 0.16 a	0.64 ± 0.02 a	4.26 ± 0.55 ab	0.31 ± 0.07 c	0.31 ± 0.07 b	0.03 ± 0.01 c	0.17 ± 0.02 a	6.04 ± 0.40 a	0.81 ± 0.17 c	6.85± 0.44 b
MeJA	1.13 ± 0.20 a	0.67 ± 0.09 a	4.71 ± 0.40 a	0.98 ± 0.15 b	1.65 ± 0.19 a	0.06 ± 0.01 b	0.10 ± 0.03 b	6.50 ± 0.60 a	2.80 ± 0.31 b	9.31 ± 0.85 a
Insect damage	0.77 ± 0.14 b	0.30 ± 0.09 b	3.44 ± 0.86 b	1.68 ± 0.31 a	1.73 ± 0.17 a	0.09 ± 0.02 a	0.12 ± 0.04 ab	4.52 ± 1.04 b	3.59 ± 0.46 a	8.11 ± 1.35 ab

For a given glucosinolate, mean values within the same column followed by the same letter are not significantly different by Student’s significance test (*p* < 0.05). ^z^ GS = glucosinolate. ^y^ Values are means of three replications.

**Table 2:** Glucosinolate hydrolysis profiles (μmol·g^−1^ DW) of “Red Russian” control kale and kale plants treated with 250 μM methyl jasmonate (MeJA) or infested with *T. ni* for 4 days ^z^.

Samples	Sulforaphane	Sulforaphane Nitrile	3-butenyl isothiocyanate	1-cyano-3,4-epithiobutane	Crambene	Goitrin	1-cyano-2-hydroxy-3,4-epithiobutane	I3A	NMI3C	NM3CA
Control	0	0.08 ± 0.02 a	0.13 ± 0.04 a	1.11 ± 0.12 a	1.48 ± 0.19 a	0	1.30 ± 0.24 a	0	0	0
MeJA	0.03 ± 0.01 a	0.11 ± 0.02 a	0.38 ± 0.04 a	1.37 ± 0.28 a	1.94 ± 0.14 a	0.07 ± 0.01 a	1.70 ± 0.21 a	0.08 ± 0.02 a	0.37 ± 0.04 a	0.05 ± 0.01a
Insect damage	0.19 ± 0.23 a	0.08 ± 0.03 a	0.75 ± 0.72 a	0.56 ± 0.28 b	1.67 ± 0.58 a	0.42 ± 0.57 a	1.28 ± 0.57 a	0.08 ± 0.03 a	0.65 ± 0.35 a	0.05 ± 0.03 a

Sulforaphane, isothiocyanate of glucoraphanin; 3-butenyl isothiocyanate, isothiocyanate of gluconapin; 1-cyano-3,4,-epithiobutane, epithioitrile from gluconapin; crambene, nitrile from progoitrin; goitrin, oxazalidine from progoitrin; 1-cyano-2-hydroxy-3,4-epithiobutane, epithioitrile from progoitrin; I3A, indolyl-3-acetonitrile, nitrile from glucobrassicin; NMI3C, *N*-methoxyindolyl-3-carbinol from neoglucobrassicin; NM3CA, *N-*methoxyindolyl-3-carboxaldehyde from neoglucobrassicin. For a given hydrolysis product, mean values within the same column followed by the same letter are not significantly different by Student’s significance test (*p* < 0.05). ^z^ Values are means of three replications.

## Supplementary Materials

Supplementary materials can be found at http://www.mdpi.com/1422-0067/19/4/1058/s1.
