# Supplementary material for: Targeted Metabolomic and Transcriptomic Analyses of “Red Russian” Kale (Brassicae napus var. pabularia) Following Methyl Jasmonate Treatment and Larval Infestation by the Cabbage Looper (Trichoplusia ni Hübner)
Source: Int J Mol Sci. 2018 Apr 2;19(4):1058. doi: 10.3390/ijms19041058 (PMC5979517; doi:10.3390/ijms19041058)

Targeted Metabolome and Transcriptome Analyses of "Red Russian" Kale (Brassicae napus var. pabularia Following Methyl Jasmonate Treatment and Larval Infestation by the Cabbage Looper (*Trichoplusia ni* Hübner)

Yu-Chun Chiu[1], John A. Juvik[2], Kang-Mo Ku[1]*

[1] Division of Plant and Soil Sciences, West Virginia University, Morgantown, WV 26506, USA; yuchiu@mix.wvu.edu (Y.-C.C.); kangmo.ku@mail.wvu.mail (K.-M.K.)

[2] Department of Crop Sciences, University of Illinois at Urbana-Champaign, Urbana, IL 61801, USA; juvik@illinois.edu (J.A.J.)

Supplementary Table S1. Glucosinolate profile of different maturity (different leaf location) of 'Red Russian' kale. Stages were separated by the different position on the plant from apical to basal leaf.

Stage	Glucoiberin	Glucoerucin	GR	Gluconapin	Progoitrin	Sinigrin	GBS	NeoGBS	4MGBS	4OHGBS	GNS
1	0.42±0.25 ab	1.94±0.44 a	7.59±1.38 a	1.40±0.52 a	47.09±9.94 a	0.37±0.14 a	8.39±3.89 a	6.79±2.33 a	0.19±0.08 a	1.52±0.46 a	1.15±0.27 a
2	0.42±0.05 a	0.67±0.19 b	5.61±0.96 b	1.51±0.76 a	24.04±8.15 b	0.16±0.05 b	3.37±1.16 b	1.74±0.61 b	0.06±0.02 b	0.46±0.08 b	0.86±0.10 a
3	0.28±0.17 ab	0.44±0.25 c	1.84±1.59 c	1.42±0.05 a	6.19±2.18 c	0.00±0.00 c	0.73±0.44 c	0.70±0.20 b	0.03±0.01 b	0.39±0.23 b	0.64±0.40 b
4	0.11±0.05 b	0.37±0.14 bc	0.25±0.15 c	0.51±0.13 b	1.91±0.43 c	0.00±0.00 c	0.17±0.13 c	0.25±0.09 b	0.00±0.00 b	0.34±0.12 b	0.38±0.20 b
5	0.35±0.21 ab	0.17±0.09 c	0.13±0.07 c	0.22±0.11 b	1.01±0.70 c	0.00±0.00 c	0.42±0.23 c	0.62±0.22 b	0.01±0.01 b	0.15±0.08 b	0.19±0.13 b

[z] Values are means of four replications. Kale leaves were harvested at five different positions (stage 1: youngest, stage 2, stage 3, stage 4, and stage 5: oldest) from the same plant to different five maturation stages[z]. Abbreviation: GR = glucoraphanin, GBS = glucobrassicin, NeoGBS = neoglubrassicin, 4MGRN = 4-methoxyglucobrassicin, 4OHBRN = 4-OH-glucobrassicin, GNS = gluconasturtiin.

Supplementary Table S2. Tentative identification of desulfo-glucosinolate in "Red Russian" kale by LC- high resolution MS in the full scan positive and negative ion mode.

t_R (min)	Trivial name	Type of side chain	DS[z] molecular formula	Measured MW^{y}_{DS}	Δ ppm	MS fragment ion (positive ionization)	MS fragment ion (negative ionization)
**7.55**	Glucoraphanin	4-methylsulfinylbutyl	$C_{12}H_{23}NO_7S_2$	358.0982	-1.927	196.02	194.92
**10.03**	Gluconapin	3-butenyl	$C_{11}H_{19}NO_6S$	294.1001	-1.341	132.05	130.03
**7.35**	Progoitrin	(2R)-2-hydroxy-3-butenyl	$C_{11}H_{19}NO_7S$	310.0948	-2.287	148.04	146.03
**15.14**	Glucobrassicin	3-indolylmethyl	$C_{16}H_{20}N_2O_6S$	369.1108	-1.743	207.06	205.04
**19.54**	Neoglucobrassicin	*N*-methoxy-3-indolylmethyl	$C_{17}H_{22}N_2O_7S$	399.1217	-0.872	237.07	235.05
**19.49**	4-methoxy-glucobrassicin	4-methoxy-3-indolylmethyl	$C_{17}H_{22}N_2O_7S$	399.1214	-1.624	237.07	235.06
**10.76**	1-hydroxy-glucobrassicin	1-hydroxy-3-indolylmethyl	$C_{16}H_{20}N_2O_7S$	385.1059	-1.371	223.05	221.04

[z]DS indicates desulfo-glucosinolate.

Supplementary Table S3. Primer set information for gene expression analysis

Gene class	Gene name	Gene model	Type	Sequence
Chain elongation	*MAM3*	Bol004799	Forward	GGTGGATGAAGAAACAGGTTACG
			Reverse	CAAACCGCCTCGATGTCTCT
	SUR1	Bol029775	Forward	GCTCCCACGTCCCGTTT
			Reverse	GCGAACCTCGAGACCACTGT
Core-structure biosynthesis	*SOT17*	Bol030757	Forward	CCATCGCCACGCTTCCT
			Reverse	CCGCCGTACTCGACGAAA
	SOT18	Bol026202v2	Forward	CCCAAAGACAGGCACCACTT
			Reverse	GGAATCGTCGAAGCGAGATC
	CYP79B2	Bol032767	Forward	GATGAAATTAAACCCACCATTAAGGA
			Reverse	GCCATGGCCCATTCGA
	SOT16	Bol039395	Forward	TTCGACGACGCCACGAA
			Reverse	CTCCACGTAAGGCACGAACTC
Secondary modification	*GSL-OH*	Bol033373	Forward	GCTTGTTGATGCTCTGTCATTGT
			Reverse	TGGCGCCGAGCGTTAG
	CYP81F1	Bol028913	Forward	CCGAGACATTCCGGCTATTC
			Reverse	CATGTCCTCCGTCGGTCTTC
	CYP81F2	Bol026044	Forward	TCTCCCACCAGGACCAACTC
			Reverse	GGTGGACCGGCGGTTT
	CYP81F3	Bol028919	Forward	CTCCTCACTCGCAACAGAATGT
			Reverse	GGAAACAAGGGCGGTTTGAT
	CYP81F4	Bol032712v2	Forward	TCCCTCTCCGCCTCACTCT
			Reverse	GGTGGACGGGAGGTTTAATGA
Transcription factors	*MYB28*	Bol036286	Forward	TCTGAGCAGATTCTCAATGAAGATG
			Reverse	TCAGGGTAAAACGTTGTTTGGA
	MYB29	Bol008849	Forward	GCTTCCATGGGCAATATCATATC
			Reverse	GACATGGAGGAGACAGTGTTGTAGA
	MYB34	Bol007760	Forward	GCTCAAACCGGTGGCAAA
			Reverse	CGTCAAGATCATCGGAGAAAGA
	MYB122	BoMYB122	Forward	CTTCCCGACAAAGCTGGACT
			Reverse	TTGGCTAAACTCACCACGCT
Myrosinase	*TGG1*	Bol017328v2	Forward	GTGCCTACGAGAGGCTATTCAAC
			Reverse	GCCGTAACATCTTTCATCAACCT
	TGG2	Bol028319v2	Forward	CGAACTCAACGCTACTGGTTACA

			Reverse	TACTCCCCTGCTCCTCTTTCC
Specifier proteins	*ESP*	Bol006378	Forward	CTACACGACTGCTACCGTCTATGG
			Reverse	GGTTGTTGGTGGGACGTTTT
	ESM1	Bol005067v2	Forward	TCCGATGTTGAACCAGTTTGC
			Reverse	CGAAGGATGGCGTTGTAGAAA
Endogenous control gene	*Actin*	Bol030974	Forward	TCCCGAGAGGAAGTACAGTGTCT
			Reverse	GAGATCCACATCTGCTGGAATG

Supplementary Table S4. Primary metabolites identified from "Red Russian" kale by GC-MS analysis.

Tentative Metabolites	Rt (min)[z]	Characteristic ion (*m*/*z*)	TMS [y]	VIP	ID[x]
Glucose	12.52	**73** [w], 147, 160, 205, 319	Meox, $(TMS)_5$	12.5	STD/ NIST[v]
Galactose	11.64	**73**, 147, *205*, 217,319	Meox, $(TMS)_5$	7.5	STD/ NIST
Sucrose	16.90	73, 147, 217, 271, **361**	$(TMS)_8$	6.3	STD/ NIST
Fructose	12.30	**73**, 130, 147, 217, 307	Meox, $(TMS)_5$	2.8	STD/ NIST
Alanine	5.65	73, **116**, 147	$(TMS)_2$	1.4	STD/ NIST

[z] Retention time.

[y] Meox, methyloxime; TMS, trimethylsilyl.

[x] Identification.

[w] Highest peak is label as bold

[v] Metabolites were identified using commercial standard compounds (STD) in comparison with the mass spectra in The National Institute of Standards and Technology (NIST) and retention time.

Supplemental Figure S1. Representative image of insect damaged kale seedlings.

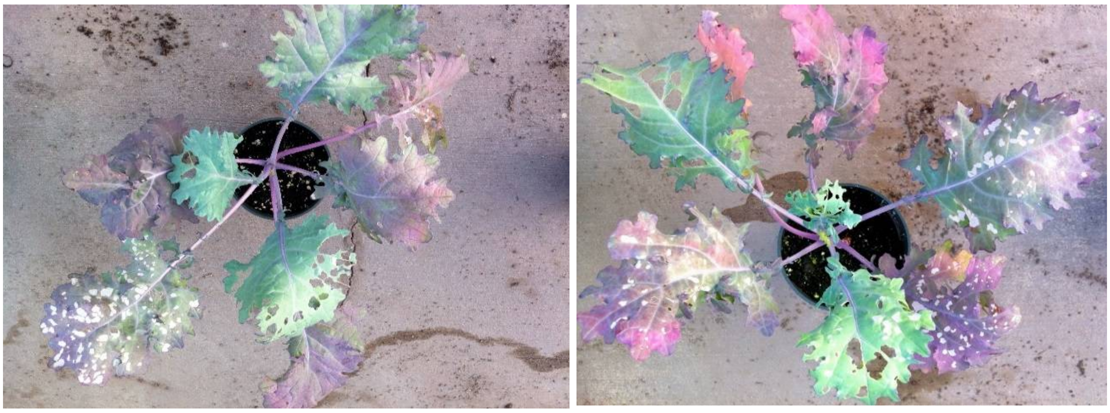

Supplemental Figure S2. Representative image of different leaf location (maturation stage) in kale seedling. (Stage 1: youngest – Stage 5: oldest).

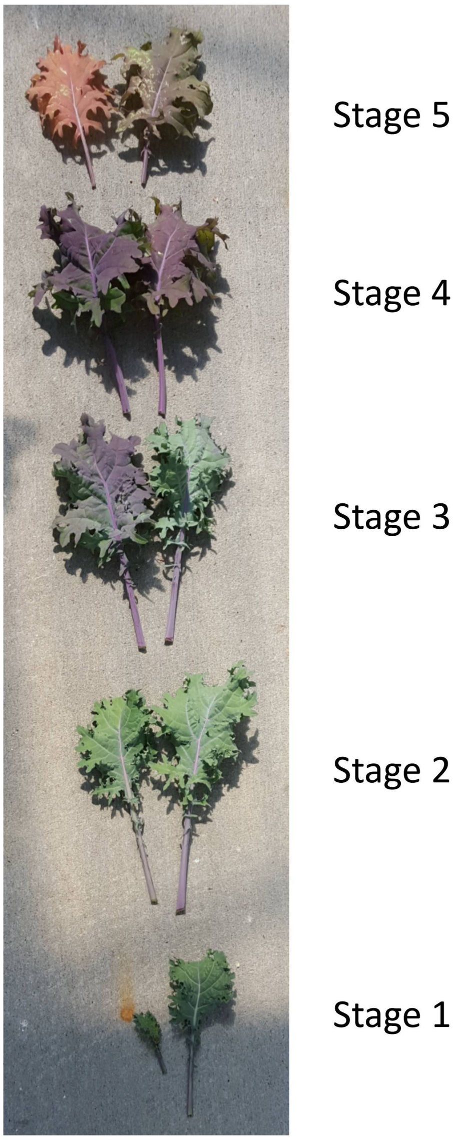

Supplement: Supplementary file 1 [file ijms-19-01058-s001.pdf]
